# Determination of Biological Activity and Biochemical Content of Ethanol Extract from Fruiting Body of *Tricholoma bufonium* (Pers.) Gillet

**DOI:** 10.3390/jof10110761

**Published:** 2024-11-02

**Authors:** Atakan Benek, Dilay Turu, Kerem Canli

**Affiliations:** 1 Department of Biology, Faculty of Science, Dokuz Eylül University, Izmir 35390, Türkiye; 2 Department of Biology, Graduate School of Natural and Applied Science, Dokuz Eylül University, Izmir 35390, Türkiye; dilayturu@gmail.com; 3 Fauna and Flora Research and Application Center, Dokuz Eylül University, Izmir 35390, Türkiye

**Keywords:** *Tricholoma bufonium*, antimicrobial activity, anti-biofilm, antioxidant activity, *Staphylococcus aureus* MRSA, *Enterococcus faecium*

## Abstract

The current study investigates the biochemical composition and biological activities of ethanol extract from the fruit body of *Tricholoma bufonium*, marking the first detailed examination of this species. The primary goal was to assess the antimicrobial, anti-biofilm, and antioxidant properties of ethanol extract from the fruit body of *T. bufonium* against a range of bacterial strains. Conventional microbiological and biochemical techniques were employed to assess the antimicrobial efficacy of the extract and to determine its minimum inhibitory concentration (MIC) and minimum bactericidal concentration (MBC) values. Furthermore, a GC-MS analysis identified bioactive compounds, such as palmitic acid and oleic acid, which are likely contributors to the observed antimicrobial activity. The anti-biofilm activity was tested using glucose monohydrate-modified environments for biofilm formation, while the antioxidant potential was measured using the DPPH radical scavenging assay, CUPRAC (cupric ion reducing antioxidant capacity) assay, and FRAP (ferric ion reducing antioxidant power) assay. The ethanol extract exhibited potent antimicrobial activity, particularly against *Enterococcus faecium*, *Bacillus subtilis*, and *Staphylococcus aureus* MRSA, with MIC values as low as 0.0338 mg/mL for several pathogens. Additionally, the extract exhibited significant anti-biofilm activity against *Bacillus cereus* and antioxidant activity with an EC_50_ value of 11.745 mg/mL. These results suggest that ethanol extract from the fruit body of *T. bufonium* may be a potent candidate for developing novel antimicrobial agents, particularly against resistant strains such as MRSA, while also providing antioxidant benefits.

## 1. Introduction

There are an estimated 1.5 million fungi worldwide, with 14,000 identified species producing fruiting bodies large enough to be classified as mushrooms [1]. Studies conducted on ethanol extracts from mushroom bodies have revealed their antidiabetic, antioxidant, anti-inflammatory, and antimicrobial activities [2,3,4]. One significant group of beneficial natural products comprises molecules synthesized by fungi. Molecules biosynthesized by fungi produce a wide range of beneficial natural compounds. Some of these natural compounds can also have strong biocidal action against human pathogenic microorganisms because of their broad-spectrum activity [5].

Antibiotics are essential medications for treating bacterial infections. The phenomenon where a bacterium can withstand the lethal or growth-inhibiting effects of an antimicrobial agent is referred to as bacterial resistance. A major contributing factor to the rise in bacterial resistance is the uncontrolled use of antibiotics. Consequently, the growing antibiotic resistance makes infections increasingly difficult or even impossible to treat with drugs that have lost their efficacy. This exacerbates the spread of infectious diseases and elevates the risk of mortality [6,7].

One of the key mechanisms that enables bacteria to develop resistance to antibiotics is the biofilm structure. Biofilm is a primary cause of chronic infectious diseases, such as infections associated with medical devices like infected catheters and surgical implants, middle ear infections, and wound infections [8]. Studies have shown that the horizontal transfer of genes that confer resistance among bacteria within the biofilm structure is 700 times more successful compared to planktonic bacterial cells [9].

The rise in antibiotic-resistant bacteria is considered a public health threat comparable to environmental and social challenges, including global warming [10]. The Centers for Disease Control and Prevention (CDC) warns that if new antibiotics are not developed by 2050, bacterial infections could cause over 10 million deaths annually, exceeding the combined fatalities from cardiovascular diseases and cancer. At present, infectious diseases are the second leading cause of death worldwide, accounting for 17 million deaths annually [11].

Given the numerous side effects and disadvantages associated with the currently used antimicrobial agents, there is an increasing utilization of bioactive substances naturally present in essential health services. In developing regions across Africa, Asia, and Latin America, over 80% of the population depends on medicinal plants for their antimicrobial properties [12]. Natural products are prolific sources of antimicrobial drugs, contributing to the development of more than half of the anticancer drugs and over half of the antibiotics currently in use [13].

The genus *Tricholoma* is commonly found in temperate and subtropical ecosystems. *Tricholoma* species are characterized by a central stipe, simple pileipellis structures, fleshy basidiomata, lamellae attached to the cap margin, white spore prints, smooth basidiospores, and, frequently, the absence of well-differentiated cystidia [14]. Biochemical content studies conducted on different species of the genus *Tricholoma* have identified the presence of compounds such as triterpenoid, D-limonene, sabinene, and cerevisterol. Additionally, it has been demonstrated that ergosterol peroxide 3-glucoside and cerevisterol are effective against human breast cancer cells [15,16,17].

To date, there is no research available on the biological activity of *T. bufonium*. The aim of this study was to examine the biological activity and biochemical composition of *T. bufonium*. The significance of this study lies in the fact that it represents the first investigation into the biological activity and biochemical content of *T. bufonium. T. bufonium* may potentially be a source of novel and effective bioactive compounds. These compounds are critically important for the development of new antimicrobial agents, particularly in light of the increasing prevalence of antibiotic resistance. Additionally, research into the medicinal use of natural products strengthens the scientific foundation of traditional medicine and contributes to the advancement of modern medicine. By elucidating the biological and chemical properties of ethanol extract from the fruit body of *T. bufonium*, this study could pave the way for new discoveries in pharmacology and microbiology, and significantly advance the field of medicinal mushroom research.

## 2. Materials and Methods

### 2.1. Collection Localities

**Figure 1:**
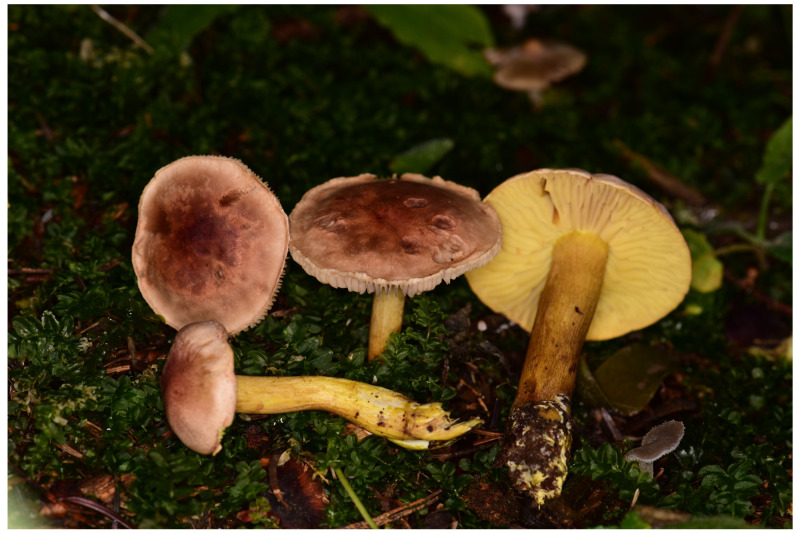
Photograph of *Tricholoma bufonium* mushroom taken during field work.

The edible mushroom species whose biological activity was investigated was selected and identified from a specimen preserved in Prof. Dr. Ilgaz Akata’s personal fungarium. The sample was collected during field studies (Figure 1) and transported to the laboratory in a sample bag. After being air-dried at room temperature, it was stored (Herbarium No: MB0018) until the experiments were conducted in the Fauna and Flora Research and Application Center (FAMER) at Dokuz Eylul University.

### 2.2. Preparation of Extracts from Tricholoma bufonium

For the extraction of active compounds, 30 g of ground (Ika, Staufen, Germany) sample was transferred into 200 mL of ethanol. The active compounds were extracted by continuous shaking at room temperature at 140 rpm (Daıhan, Wonju-si, Republic of Korea) for three days. The filtered (No:1, Whatmann, Shanghai, China) extract was then evaporated under vacuum at 35–40 °C using a rotary evaporator (R100, Buschi, Genève, Switzerland), yielding a dried mass of 0.4 g [18]. For the evaluation of antioxidant activity, the ethanol extract was diluted and prepared at a concentration of 1 mg/mL. An equivalent concentration of ascorbic acid was also prepared to serve as a positive control [19]. The solvent from the ethanol extract was fully evaporated, and the resulting residue was dissolved in distilled water containing 2% DMSO for subsequent minimum inhibitory concentration (MIC) and biofilm assays. Finally, the ethanol extract intended for GC/MS analysis was filtered through 0.45 µm injector filters to remove any remaining particles prior to analysis.

### 2.3. Determination of Antimicrobial Activity

#### 2.3.1. Microorganisms

This study employed 17 standard bacterial strains, 13 clinical isolates, 11 multidrug-resistant strains, and 7 strains isolated from food, in addition to 1 standard and 2 clinical yeast isolates. All microorganisms were sourced from the microbiology laboratory at the Department of Biology at Dokuz Eylul University.

#### 2.3.2. Microorganism Inoculum Preparation

The inocula were prepared by transferring morphologically similar colonies of each organism into a 0.9% sterile saline solution until they achieved a McFarland standard of 0.5, corresponding to approximately 1.5 × 10^8^ CFU/mL for bacteria and 1.5 × 10^7^ CFU/mL for yeasts [20].

#### 2.3.3. Testing of Antimicrobial Effect by Disk Diffusion Method

To evaluate the antimicrobial potential of the *T. bufonium* extract, the disk diffusion method was employed [21]. Sterile 90 mm Petri dishes were filled with Mueller–Hinton Agar to a precise thickness of 4.0 ± 0.5 mm. Ethanol extract volumes of 50 µL, 100 µL, and 150 µL (equivalent to 1 mg, 2 mg, and 3 mg) were applied to 6 mm sterile filter paper disks [22]. The disks impregnated with the extracts were left to dry for one day to ensure that no ethanol, which could affect the antimicrobial activity, remained on the disks. The bacterial suspension was evenly spread over the agar surface using a sterile swab. The disks containing the active ingredients were carefully placed on the inoculated agar. The bacteria were incubated (Nüve, Ankara, Türkiye) at 37 °C for 24 h, while *Candida albicans* DSMZ 1386, *Candida tropicalis*, and *Candida glabrata* were incubated at 27 °C for 48 h [23]. After the incubation phase, the widths of the inhibition zones were assessed and noted in millimeters (mm). Gentamicin and Tobramycin served as the positive control. For the negative control, disks loaded with 150 µL of pure ethanol, without any extract, were utilized.

#### 2.3.4. Testing of the Minimum Inhibitory Concentration (MIC)

The minimum inhibitory concentration (MIC) values for the *T. bufonium* DMSO–water extract were determined following the broth microdilution procedure [24]. Each strain was adjusted to match the McFarland standard of 0.5, equivalent to 1.5 × 10^8^ CFU/mL. A series of dilutions of the fungal materials were prepared and transferred into a 96-well plate. Then, microbial inoculum was added to each well. Microbial growth was examined visually. Mueller–Hinton broth (MHB) containing the test microorganisms served as the positive control. The MIC was defined as the lowest concentration of the extract needed to inhibit visible bacterial growth after 24 h of incubation at 37 °C. Results were reported in mg/mL and all experiments were performed in triplicate for precision.

#### 2.3.5. Testing of the Minimum Bactericidal Concentration (MBC)

The minimum bactericidal concentration (MBC) is defined as the lowest concentration of an antimicrobial agent that eliminates 99.9% of the initial bacterial population. After determining the MIC of *T. bufonium*, 50 µL of broth from all wells that did not exhibit visible bacterial growth was plated onto MHB agar plates and incubated at 37 °C for 24 h. The MBC was evaluated by observing the presence or absence of bacterial growth on the agar plates before and after incubation [25].

#### 2.3.6. Antibiofilm Activity

Method comprises two stages: first, establishing the conditions for biofilm formation, and second, evaluating the antibiofilm activity. The antibiofilm activity of *T. bufonium* DMSO–water extract was determined against various bacterial strains. LB broth was prepared by adding different concentrations of glucose monohydrate (0%, 0.5%, 1%, 1.5%, 2%, and 2.5%) to sterile test tubes. These were incubated at 37 °C for 24, 48, and 72 h to determine the conditions for biofilm formation. After incubation, 200 µL of crystal violet was added to each well and incubated for 15 min, followed by rinsing with distilled water (dH_2_O). In the end, each well received 200 µL of a 30:70 (*v*/*v*) acetone–ethyl alcohol solution, which was then incubated for 15 min. The ethyl alcohol solution was taken out of each well and put into the wells of a fresh plate after the incubation period. At 550 nm, the absorbance of every well was measured. For every microorganism, the ideal circumstances to produce biofilms were identified [26,27].

Sub-MIC concentrations were determined for the antibiofilm activity of the extracts in various strains. Microorganisms were adjusted to a 0.5 McFarland density. Each well of the microplates was filled with 50 µL of LB medium, 50 µL of extract, and 50 µL of strain suspension. The prepared microplates were incubated for 24, 48, and 72 h at 37 °C. The microplates were then washed with distilled water, stained with 0.1% crystal violet for 15 min, and washed again with distilled water. They were subsequently treated with a 7:3 ethanol solution for 15 min. The absorbance was measured at 550 nm, and a graph was generated using the mean values obtained from three repeated experiments.

### 2.4. Biochemical Screening

GC-MS analysis was conducted using an Agilent GC 8890-Agilent GC/MSD 5977B system (Agilent Technologies Inc., Santa Clara, CA, USA). Helium was used as carrier gas, and component identification was achieved by matching retention times with the Wiley–Nist MS data libraries. Chemical components present in quantities greater than 0.5% were considered major components. GC/MS analyses were repeated for accuracy, and some parameters were modified based on the solvents used [28].

### 2.5. Determination of Antioxidant Activity

#### 2.5.1. DPPH Radical Scavenging Method

The free radical scavenging activity of *T. bufonium* ethanol extract was assessed based on its ability to decolorize the stable DPPH radical. This method measures the conversion of the deep violet color of the DPPH solution, which is read at 515 nm, to yellow as a result of the neutralization of stable free DPPH radicals by antioxidant molecules [29]. For this assay, 0.0039 g of DPPH was dissolved in ethanol. A 96-well plate containing the DPPH solution and the extract at concentrations ranging from 7.8125 to 1000 μg/mL was incubated in the dark at room temperature for 30 min. Following incubation, the absorbance of the plate was measured at 515 nm using a microplate reader (Biotek Microplate Spectrophotometer, Shoreline, WA, USA). Ascorbic acid, a commercially known antioxidant, was used as a positive control [19]. All experiments were conducted in triplicate.

The following Formula (1) was used to calculate (%) DPPH scavenging activity.

DPPH radical scavenging (%) = [(A0 − A1)/A0] × 100(1)

where A0 is the absorbance of the DPPH solution and A1 is the absorbance of the sample.

#### 2.5.2. CUPRAC (Cupric İon Reducing Antioxidant Capacity) Method

The stock solutions consisted of a 10 mM copper (II) chloride solution prepared in distilled water, a 7.5 mM neocuproine solution prepared in methanol, and a 1 M ammonium acetate (NH_4_CH_3_CO_2_) buffer (pH = 7). After the solutions were prepared, 1 mL of copper (II) chloride solution, 1 mL of neocuproine solution, and 1 mL of ammonium acetate buffer solution were mixed in a test tube, followed by the addition of 1 mL of *T. bufonium* extract. For testing purposes, the ethanol extract was prepared by diluting it at three different concentrations: 1 mg/mL, 2.5 mg/mL, and 5 mg/mL. The mixture was incubated in the dark at room temperature for 90 mi. A yellow color was observed, indicating the reduction of copper (II) ions. The entire experiment was conducted in triplicate, with ascorbic acid used as the positive control, and 1 mL of extraction solvent, instead of the extracts, used as the negative control. Additionally, as the results were expressed in terms of ascorbic acid, a calibration curve for ascorbic acid was generated using solutions prepared at ten different concentrations (0.1–1 mM). The absorbance of the samples was measured and evaluated at 450 nm [30].

#### 2.5.3. FRAP (Ferric Ion Reducing Antioxidant Power) Method

The stock solutions consisted of a 300 mM acetate buffer solution (pH 3.6), a 10 mM TPTZ (2, 4, 6-tripyridyl-s-triazine) solution, and a 20 mM iron (III) chloride hexahydrate solution. The FRAP solution was prepared by mixing 25 mL of acetate buffer, 2.5 mL of TPTZ solution, and 2.5 mL of iron (III) chloride hexahydrate solution. For testing purposes, the ethanol extract was prepared by diluting it at three different concentrations: 1 mg/mL, 2.5 mg/mL, and 5 mg/mL. To this 2.9 mL FRAP solution, 0.1 mL of *T. bufonium* ethanol extract was added. The yellow-orange color turned into an intense blue. The mixture was incubated in the dark at room temperature for 90 min, and the results were measured at 593 nm using a microplate reader. The entire experiment was conducted in triplicate, with ascorbic acid as positive control and a mixture of 0.1 mL extraction solvent and 2.9 mL FRAP solution as the negative control. Additionally, as the results were expressed in terms of ascorbic acid, a calibration curve for ascorbic acid was generated using solutions prepared at five different concentrations (10–50 mg/L) [31].

### 2.6. Statistical Analysis

An ANOVA test was conducted to evaluate the antimicrobial activity results, with the mean inhibition zones presented alongside ± standard deviation (SD). Pearson correlation analysis was used to assess the relationship between different extract concentrations and mean inhibition results. For the antibiofilm results, conducted in nine replicates, the mean inhibition values were also reported with ± SD. The results from each antioxidant assay were reported as the mean ± SD based on three independent experimental replicates. The EC_50_ values were calculated and expressed as 95% confidence intervals using Four-Parameter Logistic Regression [32]. Data were analyzed using One-Way ANOVA (Analysis of Variance) and Pearson correlation tests in R Studio (2024.09.0+375). The significance level was set at *p* ≤ 0.05.

## 3. Results

### 3.1. Extraction Efficiency

The following Formula (2) was used to calculate extraction efficiency:

Extraction efficiency = [amount of extract (g)/amount of mushroom (g)] × 100(2)

After the extraction, the extraction efficiency was calculated and found to be 1.33%.

### 3.2. Antimicrobial Activity

The antimicrobial effectiveness of the ethanol extract derived from *T. bufonium* was evaluated against forty-five bacterial strains and three yeast strains employing the disk diffusion method. Gentamicin and Tobramycin served as positive control, while the inhibition zones around the disks containing the extract are detailed in Table 1, Table 2, Table 3 and Table 4. Disks treated with 150 µL of pure ethanol, lacking the extract, acted as negative controls. To evaluate the significance of the differences among the replicates, an ANOVA test was performed, resulting in a *p*-value of 0.925.

The Pearson correlation analysis results for the mean inhibition zones across different extract concentrations (50 µL, 100 µL, and 150 µL) for strains that showed inhibitory effects are as follows: the correlation between 50 µL and 100 µL is 0.961, between 50 µL and 150 µL is 0.921, and between 100 µL and 150 µL is 0.959. These correlation coefficients are all close to 1, indicating a strong positive correlation between the extract concentrations and the mean inhibition zones. This suggests that as the extract concentration increases, the inhibition zone also tends to increase, demonstrating a dose-dependent effect of the *T. bufonium* ethanol extract on the bacteria that showed inhibitory effects.

Ethanol extract from the fruit body of *T. bufonium* showed an inhibitory effect against 16 out of 48 strains. The strains with the highest inhibitory effects and their inhibition zone diameters are as follows: *E. faecium* (Table 3) is 18 mm, *B. subtilis* DSMZ 1971 (Table 1) is 16 mm, and *S. aureus* MRSA (Table 4) is 15 mm. These results indicate that the ethanol extract from *T. bufonium* demonstrates significant antimicrobial activity, particularly against *E. faecium*, *B. subtilis* DSMZ 1971, and *S. aureus* MRSA. These strains exhibited the highest sensitivity to the tested extract concentrations.

The minimum inhibitory concentration (MIC) values of *T. bufonium* DMSO–water extract were determined against 16 strains, as shown in Table 5. The MIC values for *T. bufonium* ranged from 4.33 to 0.0338 mg/mL. Additionally, the minimum bactericidal concentration (MBC) results, also presented in Table 5, are expressed in mg/mL, demonstrating the bactericidal effect.

The best results, indicating the lowest effective dosages, were observed for the microorganisms with the lowest MIC values for the *T. bufonium* ethanol extract. These microorganisms include *E. faecalis* ATCC 29212, *P. fluorescens* P1, *E. faecium*, and *S. flexneri*, each with an MIC value of 0.0338 mg/mL. Additionally, the MBC value for *E. faecalis* ATCC 29212 and *P. fluorescens* P1 was determined to be 4.33 mg/mL. These findings suggest that the *T. bufonium* ethanol extract is particularly effective at low dosages against *E. faecalis*, *P. fluorescens*, *E. faecium*, and *S. flexneri*. The significant antimicrobial effect observed at these low inhibitory concentrations highlights the strong antimicrobial potential of *T. bufonium* for these specific microorganisms.

### 3.3. Anti-Biofilm Activity

**Figure 2:**
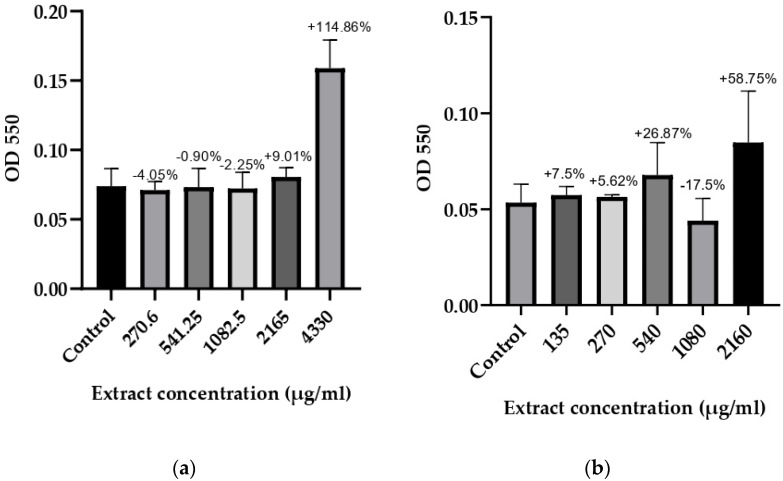
Effects of *T. bufonium* on the inhibition of biofilm formation: (**a**) *S. aureus* (CI); (**b**) *S. mutans* (CI). The medium served as the negative control. The percentage inhibition for each sample was compared with its corresponding negative control.

**Figure 3:**
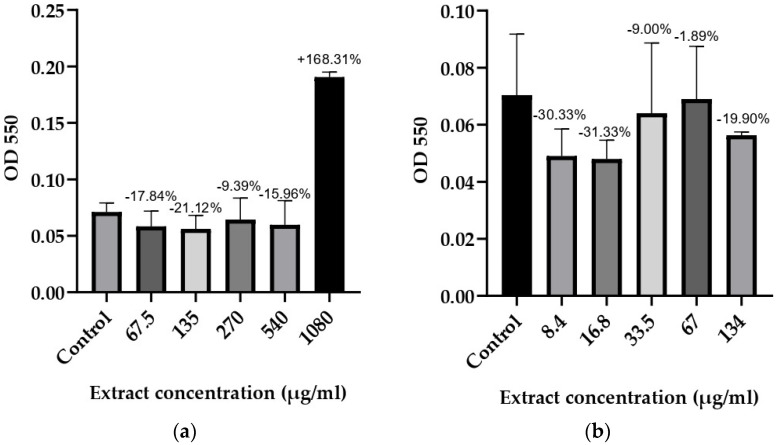
Effects of *T. bufonium* on the inhibition of biofilm formation: (**a**) *K. pneumoniae* (FI); (**b**) *S. haemolyticus* (CI). The medium served as the negative control. The percentage inhibition for each sample was compared with its corresponding negative control.

**Figure 4:**
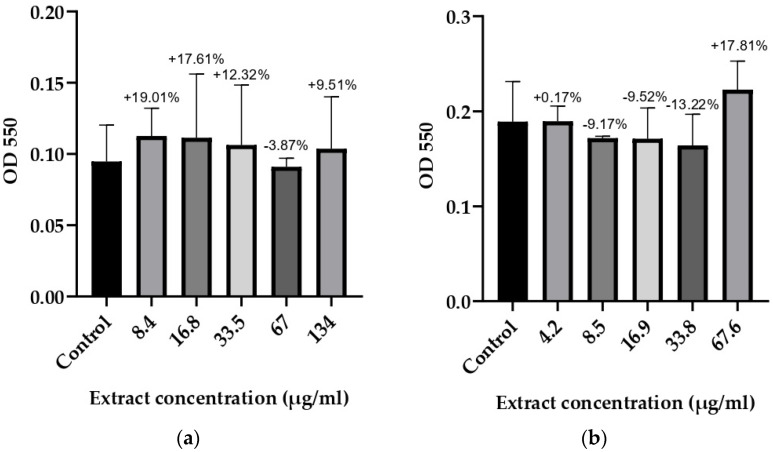
Effects of *T. bufonium* on the inhibition of biofilm formation: (**a**) *S. aureus* MRSA (MDR); (**b**) *S. hominis* ATCC 27844. The medium served as the negative control. The percentage inhibition for each sample was compared with its corresponding negative control.

**Figure 5:**
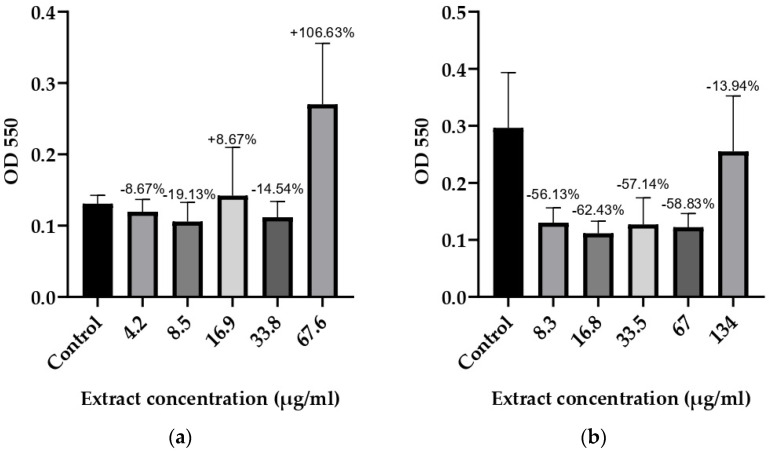
Effects of *T. bufonium* on the inhibition of biofilm formation: (**a**) *S. warneri* ATCC 27836; (**b**) *B. cereus* RSKK 863. The medium served as the negative control. The percentage inhibition for each sample was compared with its corresponding negative control.

Based on the results for the microorganisms with determined optimal biofilm conditions, it was concluded that 48 and 72 h of incubation were required at different glucose monohydrate concentrations. The anti-biofilm potential of *T. bufonium* at various concentrations was tested against eight bacterial strains, and the results are illustrated in Figure 2, Figure 3, Figure 4 and Figure 5.

All concentrations of *T. bufonium* inhibited the biofilm formation ability of *B. cereus* RSKK 863 compared to the negative control (Figure 5), although the differences among the concentrations were not statistically significant. The concentration of 16.75 µg/mL of *T. bufonium* resulted in the highest inhibition rate, achieving 62.43% ± 0.01%.

### 3.4. Antioxidant Activity

The 2,2-diphenyl-1-picrylhydrazyl (DPPH) assay was used to assess the antioxidant properties of the *T. bufonium* ethanol extract. For comparison, ascorbic acid was used as a positive control, while ethanol without the extract was used as a negative control. Table 6 displays the results pertaining to antioxidant activity. With the DPPH radical scavenging test, 11.745 mg/mL was shown to have the EC_50_ value. An ANOVA test was performed to evaluate the significance of differences between the replicates, yielding a *p*-value of 0.535. Given that the *p*-value is higher than 0.05, it may be inferred that there is no significant variation between the duplicates, indicating the consistency and dependability of the experimental results. Additionally, a substantial link was found between the extract’s concentration and its ability to scavenge DPPH radicals, as indicated by its Pearson correlation coefficient of 0.933. When the DPPH activity of the extract and ascorbic acid were compared using an additional ANOVA test, the results showed a highly significant difference (*p*-value < 2 × 10^−16^). Furthermore, the EC_50_ value of ascorbic acid was determined to be 0.04 mg/mL, with the 95% confidence interval calculated using Four-Parameter Logistic Regression.

The CUPRAC reduction result of the *T. bufonium* extract is presented as the ascorbic acid equivalent (AAE) in mM in Table 7. Since the results will be evaluated in terms of ascorbic acid, the calibration curve (y = 3.7723x + 0.1535, R^2^ = 0.9904) was drawn. The ethanol extract from *T. bufonium* demonstrated strong antioxidant activity across various concentrations. The means (±SD) are reported with their standard deviations. All values increased with higher concentrations (1.0–5.0 mg/mL) of the mushroom extract. The correlation between concentrations and AAE (mM) exhibited a strong positive relationship (R^2^ = 0.99866). A statistically significant difference (*p* < 0.05) was observed at all tested concentrations of the ethanol extract from *T. bufonium*.

The reducing ability of the ethanol extract from the mushroom was expressed as ascorbic acid equivalents (AAEs) in mg/L of extract, as shown in Table 8. Since the results will be evaluated in terms of ascorbic acid, the calibration curve (y = 0.0144x + 0.145, R^2^ = 0.9998) was drawn. *T. bufonium* exhibited strong antioxidant activity across different concentrations. The values increased consistently with higher concentrations (1.0–5.0 mg/mL) of the mushroom extract. A strong positive correlation (R^2^ = 0.994334) was observed between the concentrations and AAE (mg/L). A statistically significant difference (*p* < 0.05) was found at all tested concentrations of the ethanol extract from *T. bufonium*.

### 3.5. Chemical Composition Analysis

The chemical composition of the ethanol extract from *T. bufonium* was analyzed using GC/MS (Gas Chromatography–Mass Spectrometry). The compounds constituting more than 0.5% of the extract were classified as major components. Table 9 presents these primary components, providing information on the chemical structure, chemical formula, molecular weight, and known activities of each compound.

## 4. Discussion

The tests conducted to determine antimicrobial activity revealed that *T. bufonium* extract exhibited the highest effect against the food isolate *E. faecium* strain. *E. faecium*, a Gram-positive bacterium, can tolerate extreme conditions such as high salt concentrations and is commonly found in raw or fermented foods [33]. Although studies indicate that *E. faecium* strains are used in food production, they have been shown to possess virulent factors and genes associated with pathogenicity [34]. *E. faecium* strains are known to cause wound and surgical site infections, bloodstream infections, urinary tract infections, endocarditis, and diarrhea. Therefore, the use of *E. faecium* as a probiotic raises significant concerns [35]. The European Food Safety Authority (EFSA) has stated that enterococci do not meet the “Qualified Presumption of Safety” criteria and has confirmed the pathogenic potential of *E. faecium* [36]. Considering all these factors, the effect exhibited by the *T. bufonium* extract suggests that it could be a potential candidate for cases where, in the future, antibiotics prove insufficient in treating *E. faecium*-related infections.

The *T. bufonium* ethanol extract was found to be effective against six out of eleven *Staphylococcus* strains. Notably, the extract showed activity against the *S. aureus* MRSA strain. *S. aureus* MRSA strains possess the ability to produce penicillin-binding proteins (PBP) in response to semi-synthetic penicillin. By producing PBP, *S. aureus* MRSA strains can resist the effects of many commonly used antibiotics. Infections caused by *S. aureus* MRSA can affect the heart, bones, lungs, and circulatory system of patients. The World Health Organization (WHO) has listed *S. aureus* MRSA as a “priority pathogen.” Therefore, the activity of the *T. bufonium* extract against the *S. aureus* MRSA strain is of great importance [37]. The GC-MS analysis identified palmitic acid as a component of the biochemical composition of *T. bufonium*. Previous studies have demonstrated the antimicrobial effects of palmitic acid, further supporting the antimicrobial activity exhibited by the *T. bufonium* ethanol extract [38].

Another significant effect observed among the results was against the *S. mutans* strain. *S. mutans* plays a key role in the development of dental caries [39]. By adhering to the surface of teeth, *S. mutans* facilitates the attachment and colonization of various microorganisms in the oral cavity. It produces glucosyltransferases, which mediate the synthesis of exopolysaccharides called glucans, which help in the formation of acid-producing biofilms, leading to dental plaque formation and tooth demineralization [40,41]. Oral microorganisms are closely associated with various oral diseases such as dental caries, periodontal disease, and oral cancer [42]. Traditional antibiotic therapy has demonstrated limited efficacy in the clinical management of *S. mutans* infections, underscoring the urgent need for innovative antimicrobial and antibiofilm strategies [43]. Therefore, the effect of the *T. bufonium* extract against *S. mutans*, a key player in oral disease development, is of great significance. The GC-MS analysis revealed the presence of oleic acid in the biochemical composition of *T. bufonium*. Oleic acid is known for its bactericidal effect against streptococci, which further supports the antimicrobial effect of the *T. bufonium* extract against *S. mutans* [44].

*B. cereus* is known to cause foodborne infections and is considered an etiological agent of vomiting and diarrhea syndromes [45]. The vegetative cells of *B. cereus* can survive and proliferate at pH values ranging from 5 to 10. *B. cereus* spores are highly resistant to extreme conditions such as high temperatures, freezing, drying, and gamma and UV radiation, which allows *B. cereus* to survive on various surfaces. Biofilm production enables *B. cereus* to thrive in challenging environments [46]. In particular, carbon sources, minerals, and food residues found in areas where food production occurs can significantly influence biofilm formation by *B. cereus* [47]. Taking all of this into account, the inhibitory effect of *T. bufonium* extract against *B. cereus*, which poses a serious problem that needs to be eliminated in biofilm structures, holds promise for the future. It is known that pentadecanoic acid present in the biochemical composition of *T. bufonium* has an inhibitory effect on the biofilm structure of *K. pneumoniae* strains [48]. The known biofilm inhibitory effect of pentadecanoic acid supports the observed biofilm inhibition against *B. cereus*.

This study is the first to demonstrate the antioxidant activity of *T. bufonium* extract. The antioxidant activities of *Tricholoma matsutake* and *Tricholoma terreum*, species belonging to the genus *Tricholoma*, have been assessed using the DPPH method, which was also utilized in this study. In another study, the antioxidant activity of *T. matsutake* extract was determined, and it was found to have a 68.74% radical scavenging rate at a concentration of 800 µg/mL. In another study, the ethanol and methanol extracts of *T. terreum* were tested, and the IC_50_ values were found to be 12.17 ± 0.03 and 30.45 ± 0.12 mg/mL, respectively. These studies indicate that the *Tricholoma* genus exhibits antioxidant effects. Squalene, a compound identified in the biochemical structure of *T. bufonium*, is known to possess antioxidant properties, further supporting the analyzed antioxidant activity [49,50,51].

Antioxidant activities of certain species belonging to the genus Tricholoma have been demonstrated using various methods. In a study examining the antioxidant activity of *T. caligatum* and *T. columbetta* species’ cyclohexane, dichloromethane, methanol, and water extracts via the FRAP method, the highest result was obtained from the water extract from *T. caligatum* with 17.29 ± 0.38 μM TE/g. In this study, the antioxidant capacity of *T. bufonium* could not be compared with *T. caligatum* and *T. columbetta* because the results obtained for antioxidant capacity were measured in different units. However, both studies indicate that species belonging to the genus Tricholoma exhibit antioxidant activity [52].

In conclusion, palmitoleic acid, identified through GC-MS analysis, has emerged as a significant biomarker of health, with essential roles in metabolic regulation. It enhances insulin sensitivity, promotes β cell proliferation, and reduces endoplasmic reticulum stress in various studies. These complexities underscore the need for further research to elucidate palmitoleic acid’s mechanisms and implications for metabolic diseases, potentially paving the way for innovative therapeutic strategies [53]. *T. bufonium* ethanol extract could serve as a valuable resource for similar studies.

## 5. Conclusions

The studies in the literature regarding *T. bufonium* primarily focus on its flora. This research presents, for the first time, the biochemical composition and biological activities of the *T. bufonium* species. The tests conducted indicate that *T. bufonium* exhibits biological activity, and with future advanced studies, the observed effects can be further elucidated, potentially uncovering additional effects of the species.

## Figures and Tables

**Table 1:** Antimicrobial susceptibility of standard isolated strains by disk diffusion method.

Strains	50 μL	100 μL	150 μL	Gentamicin (10 µg)	Tobramycin (10 µg)
*Acinetobacter baumannii* CECT 9111	-	-	-	13 ± 0.00	22 ± 0.00
*Bacillus cereus* RSKK 863	10 ± 0.00	10 ± 0.57	12 ± 0.00	24 ± 0.00	18 ± 0.00
*Bacillus subtilis* DSMZ 1971	12 ± 0.00	14 ± 0.00	16 ± 0.00	30 ± 0.00	26 ± 0.00
*Candida albicans* DSMZ 1386	-	-	-	12 ± 0.00	13 ± 0.00
*Enterobacter aerogenes* ATCC 13048	-	-	-	24 ± 0.00	18 ± 0.00
*Enterococcus faecalis* ATCC 29212	7 ± 0.00	8 ± 0.00	9 ± 0.57	12 ± 0.00	8 ± 0.00
*Escherichia coli* ATCC 25922	-	-	-	22 ± 0.00	20 ± 0.00
*Listeria monocytogenes* ATCC 7644	-	-	-	28 ± 0.00	24 ± 0.00
*Pseudomonas aeruginosa* DSMZ 50071	-	-	-	15 ± 0.00	22 ± 0.00
*Pseudomonas fluorescens* P1	-	7 ± 0.00	8 ± 0.00	13 ± 0.00	12 ± 0.00
*Salmonella enteritidis* ATCC 13076	-	-	-	21 ± 0.00	20 ± 0.00
*Salmonella typhimurium* SL 1344	-	-	-	24 ± 0.00	15 ± 0.00
*Shigella flexneri* RSKK 184	-	-	-	18 ± 0.00	17 ± 0.00
*Staphylococcus aureus* ATCC 25923	-	-	-	21 ± 0.00	14 ± 0.00
*Staphylococcus epidermidis* DSMZ 20044	10 ± 0.00	10 ± 0.00	11 ± 0.57	22 ± 0.00	20 ± 0.00
*Staphylococcus hominis* ATCC 27844	10 ± 0.00	11 ± 0.00	12 ± 0.00	18 ± 0.00	16 ± 0.00
*Staphylococcus warneri* ATCC 27836	9 ± 0.00	11 ± 1.00	13 ± 0.00	23 ± 0.00	18 ± 0.00

(-) no inhibition, the values are reported as mean ± standard deviation (n = 3), reflecting the results of three independent experiments that produced consistent findings.

**Table 2:** Antimicrobial susceptibility of clinic isolated strains by disk diffusion method.

Strains	50 μL	100 μL	150 μL	Gentamicin (10 µg)	Tobramycin (10 µg)
*Acinetobacter baumannii*	-	-	-	18 ± 0.00	16 ± 0.00
*Candida glabrata*	-	-	-	7 ± 0.00	8 ± 0.00
*Candida tropicalis*	-	-	-	-	-
*Enterococcus faecalis*	-	-	-	12 ± 0.00	10 ± 0.00
*Klebsiella pneumoniae*	-	-	-	18 ± 0.00	18 ± 0.00
*Shigella boydi*	-	-	-	20 ± 0.00	18 ± 0.00
*Shigella flexneri*	-	-	7 ± 0.00	16 ± 0.00	14 ± 0.00
*Staphylococcus aureus*	-	-	-	22 ± 0.00	18 ± 0.00
*Staphylococcus aureus*	-	-	9 ± 0.00	22 ± 0.00	16 ± 0.00
*Staphylococcus haemolyticus*	-	7 ± 0.00	8 ± 0.00	10 ± 0.00	10 ± 0.00
*Staphylococcus hominis*	-	-	-	9 ± 0.00	11 ± 0.00
*Staphylococcus lugdunensis*	-	-	-	17 ± 0.00	18 ± 0.00
*Streptococcus mutans*	11 ± 0.00	13 ± 0.57	14 ± 0.00	22 ± 0.00	24 ± 0.00

(-) no inhibition, the values are reported as mean ± standard deviation (n = 3), reflecting the results of three independent experiments that produced consistent findings.

**Table 3:** Antimicrobial susceptibility of food isolated strains by disk diffusion method.

Strains	50 μL	100 μL	150 μL	Gentamicin (10 µg)	Tobramycin (10 µg)
*Enterococcus durans*	9 ± 0.00	11 ± 0.00	13 ± 0.00	11 ± 0.00	13 ± 0.00
*Enterococcus faecium*	16 ± 0.00	18 ± 0.57	18 ± 0.57	28 ± 0.00	15 ± 0.00
*Escherichia coli*	-	-	-	-	-
*Klebsiella pneumoniae*	-	-	-	19 ± 0.00	23 ± 0.00
*Listeria innocua*	-	-	-	13 ± 0.00	15 ± 0.00
*Salmonella infantis*	-	-	-	17 ± 0.00	14 ± 0.00
*Salmonella kentucky*	-	-	-	12 ± 0.00	16 ± 0.00

(-) no inhibition, the values are reported as mean ± standard deviation (n = 3), reflecting the results of three independent experiments that produced consistent findings.

**Table 4:** Antimicrobial susceptibility of multi-drug resistance strains by disk diffusion method.

Strains	50 μL	100 μL	150 μL	Gentamicin (10 µg)	Tobramycin (10 µg)
*Achromobacter* sp.	10 ± 0.00	11 ± 0.00	12 ± 0.00	9 ± 0.00	-
*Acinetobacter baumannii*	-	-	-	-	-
*Enterobacter aerogenes*	-	-	-	16 ± 0.00	18 ± 0.00
*Escherichia coli*	-	-	-	8 ± 0.00	9 ± 0.00
*Klebsiella pneumoniae*	8 ± 0.00	9 ± 0.00	10 ± 0.00	15 ± 0.00	20 ± 0.00
*Proteus vulgaris*	-	-	-	11 ± 0.00	11 ± 0.00
*Providencia rustigianii*	-	-	-	16 ± 0.00	19 ± 0.00
*Serratia odorifera*	-	-	-	7 ± 0.00	9 ± 0.00
*Staphylococcus aureus MRSA*	11 ± 0.57	14 ± 0.57	15 ± 0.00	-	7 ± 0.00
*Staphylococcus aureus MRSA+MDR*	-	-	-	22 ± 0.00	21 ± 0.00
*Streptococcus pneumoniae*	-	-	-	10 ± 0.00	8 ± 0.00

(-) no inhibition, the values are reported as mean ± standard deviation (n = 3), reflecting the results of three independent experiments that produced consistent findings.

**Table 5:** Minimum inhibitory concentration (MIC) and minimum bactericidal concentration (MBC) values for *T. bufonium*.

Strains	MIC (mg/mL)	MBC (mg/mL)
*Achromobacter* sp.	-	-
*Bacillus cereus* RSKK 863	0.134	-
*Bacillus subtilis* DSMZ 1971	-	-
*Enterococcus durans*	4.33	-
*Enterococcus faecalis* ATCC 29212	0.0338	4.33
*Enterococcus faecium*	0.0338	-
*Klebsiella pneumoniae*	1.08	-
*Pseudomonas fluorescens* P1	0.0338	4.33
*Shigella flexneri*	0.0338	-
*Staphylococcus aureus*	4.33	-
*Staphylococcus aureus* MRSA	0.134	-
*Staphylococcus epidermidis* DSMZ 20044	0.0676	-
*Staphylococcus haemolyticus*	0.134	-
*Staphylococcus hominis* ATCC 27844	0.0676	-
*Staphylococcus warneri* ATCC 27836	0.0676	-
*Streptococcus mutans*	2.16	-

(-) no inhibition.

**Table 6:** DPPH radical scavenging activity of *T. bufonium* ethanol extract and ascorbic acid (%) with mean ± SD.

Concentration (µg/mL)	DPPH Radical Scavenging Activity of *T. bufonium (%)*	DPPH Radical Scavenging Activity of Ascorbic Acid (%)
1000	38.02 ± 1.21	94.66 ± 0.02
500	27.98 ± 0.41	93.39 ± 0.06
250	24.72 ± 1.63	92.07 ± 0.11
125	20.49 ± 0.04	90.08 ± 0.05
62.5	19.33 ± 0.00	69.94 ± 0.05
31.25	17.65 ± 1.65	35.79 ± 0.08
15.625	12.76 ± 0.02	17.69 ± 0.19
7.81	9.91 ± 0.69	8.739 ± 0.18

**Table 7:** CUPRAC cupric ion reducing antioxidant capacity of *T. bufonium* ethanol extract and ascorbic acid (%) with mean ± SD.

Concentration of *T. bufonium* Ethanol Extract (mg/mL)	Ascorbic Acid Equivalent (AAE) mM
5	0.94 ± 0.05
2.5	0.54 ± 0.01
1	0.26 ± 0.00

**Table 8:** FRAP ferric ion reducing antioxidant activity of *T. bufonium* ethanol extract and ascorbic acid (%) with mean ± SD.

Concentration of *T. bufonium* Ethanol Extract (mg/mL)	Ascorbic Acid Equivalent (AAE) mg/L
1	29.79 ± 1.39
2.5	52.99 ± 1.59
5	80.86 ± 2.00

**Table 9:** GC-MS analysis of *T. bufonium* ethanol extract components.

Peak	RT	Area (%)	Compound	Formula	MW (g/mol)
1	32.636	1.45	Pentadecanoic acid	C_15_H_30_O_2_	242.40
2	36.001	6.31	Palmitoleic Acid	C_16_H_30_O_2_	254.41
3	37.107	18.48	Palmitic Acid	C_16_H_32_O_2_	256.42
4	38.341	1.18	Unkown	-	-
5	41.439	7.01	Oleic acid	C_18_H_34_O_2_	282.5
6	42.039	1.22	Stearic Acid	C_18_H_36_O_2_	284.5
7	42.698	1.01	Methoxyacetic acid, 2-tetradecyl ester	C_17_H_34_O_3_	286.4
8	47.304	1.2	Unknown	-	-
9	50.597	9	Phthalic acid, 2-ethylhexyl isohexyl ester	C_22_H_34_O_4_	362.5
10	54.996	4.51	Bis(2-ethylhexyl) terephthalate	C_24_H_38_O_4_	390.6
11	56.895	44.67	Squalene	C_30_H_50_	410.7
12	57.488	1.74	Cholesta-3,5-diene	C_27_H_44_	368.4
13	57.981	1.02	Unknown	-	-
14	60.163	1.2	Unknown	-	-

(-) unknown; RT: retention time; MW: molecular weight.

## Data Availability

The original contributions presented in the study are included in the article material, further inquiries can be directed to the corresponding authors.
